# The Impact of Structural Determinants on Pregnancy Intention and Reproductive Decision-Making Among African American Women

**DOI:** 10.1007/s40615-025-02459-w

**Published:** 2025-05-19

**Authors:** Jill Strickland Denson

**Affiliations:** Prevention Research Center, University of Wisconsin, Madison, WI USA

**Keywords:** Group concept mapping, Reproductive decision-making, Structural determinants of health, Racial disparities in pregnancy outcomes, African American women, Code: 91 C99

## Abstract

**Introduction:**

African American women face significant reproductive health inequities rooted in structural and social factors, though few studies have directly engaged them to examine how such factors play a role in their reproductive decision-making. This study draws from a methodology underused in reproductive health research to understand how African American women of different socioeconomic groups approach reproductive decision-making.

**Methods:**

The study employed group concepting mapping (GCM), a participatory, mixed-methods design that integrates qualitative processes with multivariate analyses. Twenty-four African American women between 18 and 44 years were recruited into lower (*n* = 12) and higher (*n* = 12) income groups. Based on GCM methods, each group engaged in the participatory data collection analysis process across three sessions. GCM software was used to generate visualizations, such as pattern match maps and go-zones, to identify themes.

**Results:**

Participants described experiences with trauma, depression, stress, and everyday interaction with discrimination. Three themes emerged from the GCM process: inward reflection (autonomy in reproductive decision-making, prioritizing career and education, and pressure to push through adversity), relational reflection (expectations of sexual partners in reproductive trajectories), and outward reflection (judgement from providers, reproductive coercion in healthcare systems). Each theme influenced participants’ pregnancy trajectories and reproductive decision-making.

**Conclusions:**

Participants reported high importance of self-reflection, career and education, and low importance of male partners, when considering whether to pursue pregnancy, prevent pregnancy, and/or use contraception. Findings illustrate participants’ reproductive health experiences were impacted by structural and social determinants. Results help document opportunities for policies and practices to attend to structural determinants versus individuals’ reproductive trajectories.

## Introduction

Unintended pregnancy is an unwanted or mistimed conception and is associated with poor maternal health outcomes, and disparities in unintended pregnancies are linked with a cycle of disadvantage for underserved populations [1–3]. Nearly half of all pregnancies in the USA are unintended, with the majority of these unintended pregnancies experienced by African American women [4–9]. African Americans have 2.3 times the infant mortality rate as non-Hispanic Whites. African American infants are 3.8 times as likely to die from complications related to low birth weight as compared to non-Hispanic White infants [10]. African American women are 2 to 3 times more likely to die from pregnancy related causes than White women, and the disparity increases with age [11]. Discrimination based on multiple social identities may play a role in disparities in pregnancy outcomes.

Recently, scholars have begun to directly investigate discrimination based on multiple social identities through intersectional frameworks [12, 13]. Social determinants of health have broadly become accepted as a legitimate public health construct; however, there is less emphasis in science and practice on the potential link between historical racism and trauma and poor outcomes across generations. This is especially evident in the fields of sexual and reproductive health, and maternal and child health, where in the USA, unintended pregnancy and infant and maternal mortality rates are the highest for the African American population [14–17]. Although there are studies that focus on the reproductive health of African American women, few studies specifically examine the role of racism in sexual and reproductive health [18].

This study builds upon scholarship in historical trauma and racism that examined tropes about African American women’s sexuality, historical traumas, relationship to the health care system, and the intersection of race and gender-based discrimination, focusing on race, gender, and class [12, 13, 19–27]. Public health scholars have been less apt to study individuals and communities using an intersectional lens; public health scholars routinely examine systems independently. To better understand racism as a public health issue, there is a need for greater application of intersectionality to public health frameworks to examine multiple and interwoven structural influences that negatively affect people of color, especially African American women. This study addresses pregnancy intention in African American women and the problematic nature of health care and research on this topic due to a lack of accounting of the complexities of identity and structural racism in the explanation of disparities in birth outcomes and pregnancy intention. This study used group concept mapping (GCM) to understand how African American women of different socioeconomic groups engage with reproductive decision -making.

To move past disparity research comparing African American and White women, this study centered African American women to learn more about their intersecting social identities and how they navigated reproductive decision-making. It was important to use a qualitative method to understand how African American women make reproductive decisions in their own words.

## Methodology

GCM is a participatory mixed-methods design, which has been used in behavioral science and public health research to research topics including health disparities, infant mortality, cancer, physical health, and intimate partner violence and integrates qualitative processes, such as focus group-like sessions of 10 to 40 participants, with multivariate analyses [29–31]. The strength of this method for this study is the participatory nature, where participants’ voices are present throughout data collection, analysis, and emergence of findings. The aim is to capture ideas generated from participants’ lived experiences and create a visual map of ideas discussed. All participants have an equal voice and contribute to the visual map that represents the statements generated and how they relate to each other as well as rating the importance of the statements. GCM provides the ability to capture and represent participant ideas in a group setting in a series of related two-dimensional maps [32]. This study used GCM because of its participatory nature throughout the entire process of data collection and interpretation that centers participants as the experts in their experiences, and the ability to turn the ideas of the participants into visual outputs (point maps, cluster maps, and pattern matches) the participants view, use for decision-making (e.g., which cluster map is the best fit), and interpret together in group sessions [31]. GCM can address questions where the knowledge generated by participants guide the research priorities [32]. GCM is a stronger approach for understanding complex phenomena, such as reproductive decision-making among African American women, grounded in intersectionality and critical race theory, than focus groups or interviews alone. It includes the multidimensional scaling of the statement by the software to visualize the relationships between ideas in point maps, cluster maps, and pattern matches, which more traditional qualitative methods do not. GCM sessions took place in a mid-sized city in Wisconsin between September 9 and September 23, 2019. The IRB at the researcher’s university approved this study.

### Participants

Participants for this study were included if they met the self-reported criteria of being African American, female at birth, able to conceive at some point during their lifetime, ages 18–44 years (reproductive age), a resident of the county where the study took place, non-immigrant, and had an annual income up to 400% of the federal poverty level (FPL) (2019 guidelines) [33].

Participants in this study were recruited through informational flyers disseminated at local health systems, federally qualified health centers, WIC offices, planned parenthood clinics, universities, grocery stores, and other public locations, as well as through the researcher’s electronic networks and social media, and assigned to a group.

Interested participants were invited to attend one of two groups depending on their income: the lower-income group included those whose gross annual household income was 0–250% FPL and the higher-income group included those whose gross annual household income was 251% or greater of FPL [33]. Table 1 displays demographics of the two groups.

**Table 1:** Sample demographics grouped by lower or higher income

Characteristic	Lower-income group		Higher-income group	
Sample size	12		12	
Education level	*N*	%	*N*	%
Some high school or less	3	25.0	0	0.0
Completed high school or obtained GED	4	33.3	1	8.3
Trade school	1	8.3	0	0.0
Some college	0	0.0	1	8.3
Associate degree or 2-year college degree	1	8.3	1	8.3
Bachelor’s degree or 4-year college degree	2	16.7	5	41.7
Master’s degree or higher	1	8.3	4	33.3
Age range	*N*	%	*N*	%
18–20 years	3	25.0	0	0.0
21–29 years	4	33.3	3	25.0
30–39 years	3	25.0	6	50.0
40–44 years	2	16.7	3	25.0
Relationship status	*N*	%	*N*^1^	%
Single, never married	7	58.3	4	36.4
Married	0	0.0	2	18.2
Living with a partner	3	25.0	3	27.3
Separated	1	8.3	0	0.0
Divorced	0	0.0	2	18.2
Widowed	1	8.3	0	0.0
Type of medical coverage	*N*	%	*N*	%
Private insurance from employer	1	8.3	12	100.0
BadgerCare Plus HMO, or Forward Health Card	4	33.3	0	0.0
Medicaid or T19	2	16.7	0	0.0
Medicaid or T19 SSI	2	16.7	0	0.0
Exchange, or ACA/Obamacare	1	8.3	0	0.0
Unsure of health insurance coverage type	2	16.7	0	0.0
Annual household income	*N*	%	*N*	%
Less than $9,999	6	50.0	0	0.0
$10,000–$19,999	2	16.7	0	0.0
$20,000–$29,999	3	25.0	1	8.3
$30,000–$39,999	0	0.0	1	8.3
$40,000–$49,999	1	8.3	2	16.7
$50,000–$59,999	0	0.0	2	16.7
$60,000–$69,999	0	0.0	1	8.3
$70,000–$79,999	0	0.0	1	8.3
$80,000–$89,999	0	0.0	0	0.0
$90,000–$99,999	0	0.0	2	16.7
$100,000 or more	0	0.0	2	16.7

^1^Eleven of 12 respondents answered the relationship status question from the higher-income group

### Procedure

The GCM process was implemented across three sessions for each group, and participants were invited to attend all three sessions. Each session had specific deliverables related to GCM that are described in detail in Table 2. Participants were encouraged to attend all three sessions as each concept mapping session builds on the previous session’s work. Participants were compensated for their time through gift cards and bus tickets and gas cards for transportation. Data completion was incentivized, compensation for session one was $15, session two was $25, and session three was $35 for a total of $75 for completing all sessions.

**Table 2:** Group concept mapping steps

GCM step	Explanation
GCM Step 1: Preparation	In preparation for the GCM participant sessions, the researcher developed a prompt that would be used in the brainstorming activity with the women in the first group session. The researcher and trained research assistant established their roles in each GCM session and developed the instructions for the brainstorming activity in the first GCM session
GCM Step 2: Brainstorming	In session 1, the researcher provided an orientation to the study and participants completed a consent form. The researcher provided a focus prompt to begin this brainstorming session: “What are the factors that you consider when deciding to become pregnant or not?” During this brainstorming activity, participants generated a total of 102 statements between both the lower-income and higher-income groups. Following session 1, a research assistant entered into the GCM software all of the statements generated from this brainstorming step
GCM Step 3: Sorting and rating	In session 2, participants were asked to complete two activities. First, they independently sorted the statements generated from the first session. Each participant was given a stack of 102 notecards. Each notecard contained a statement generated from the brainstorming session. The participants read the statement on each card and sorted them into piles that reflected similar ideas. They assigned each pile a name that reflected their understanding of how the statements in the pile related to each other. They could create as many or as few piles from the statements as they believed fit the statements. The research assistant entered into the GCM software each individual participants’ results of the sorting session, including how they named each pile and the statements they categorized within each pile After the sorting process, the participants completed a rating process. Each participant received three rating sheets that asked the participants to rate the importance of each of the 102 statements on a 5-point Likert scale, where 1 meant “relatively unimportant” and 5 meant “extremely important.” The three rating questions were (1) How important is each factor in deciding to get pregnant?; (2) How important is each factor in preventing pregnancy?; (3) How important is each factor in determining if you will use birth control? The research assistant recorded the ratings from the rating sheets that each participant submitted into the GCM software. See Fig. 1 for an example rating sheet
GCM Step 4: Creation and interpretation of maps/representation and interpretation	The GCM software generated a point map based on the participants’ sorting and ranking of the 102 statements generated in session one, where each point on the map is a representation of each statement, and the relative position of each point or statement is representative of how related the points or statements are to one another, based on participant sorting of statements. Points or statements that are closer together on the map have been more frequently sorted together by participants than points or statements that are spatially further apart on the map. Cluster analysis is the second visualization part of the GCM process where individual statements on the map are placed into clusters of statements with similar concepts, creating a cluster map illustrating how the multidimensional scaling points were grouped [30, 31]. The GCM software program from the Concept System Global MAX analysis was used for hierarchical cluster analysis employing Ward’s algorithm in the *x*–*y* coordinate data obtained from multidimensional scaling as the standard procedure, effectively partitioning the map produced via multidimensional scaling into any number of hierarchical clusters. At each stage in the analysis, the algorithm combines two clusters until, at the end, all the statements are in a single cluster [31]. This took place following session two so the preliminary clusters generated by the software from the ratings in session 2 could be reviewed by participants in session three Session 3 was a participatory analysis session where participants reviewed the preliminary point map and cluster maps generated by the GCM software and decided whether each statement and each cluster were representative of what they were thinking as they generated and rated the statements in previous sessions. Further, the GCM software generated multiple preliminary cluster map scenarios, each with statements grouped into a different number of clusters. The participants reviewed the point map and each different cluster scenario generated by the software. The participants selected the number of clusters that best matched their understanding of the sorting and rating of the statements, eliminated redundancies and any statements that were inaccurate based on their understanding of each map. The groups decided how the clusters should be named to most closely match the meaning they ascribed to the statements sorted within that cluster. After the participants were confident the cluster map with eight clusters represented their ideas accurately, the researcher was able to use the GCM software to select this cluster map and use this to generate additional visualizations: pattern match maps and go-zones. These did not need to be done in order. These visualizations allowed the researcher to compare responses between groups based on income level

**Fig. 1:**
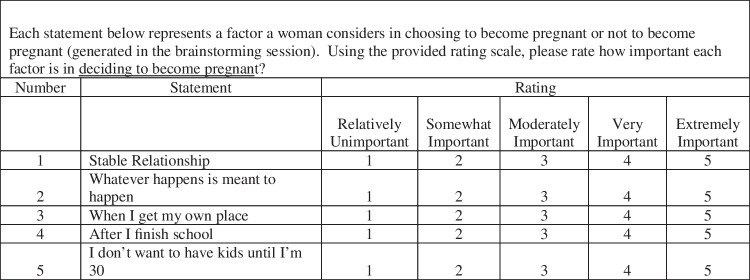
Example rating sheet for rating Question 1

### GCM Activities

The GCM process included preparation, brainstorming, sorting and rating activities, and creation and interpretation of maps, or representation and interpretation. See Table 2 for detailed steps of this process.

## Results

Twenty-four participants were included in this study’s sample with 12 in each income group. Demographic data were collected on education level, age range, relationship status, health insurance type, and income. Seven participants (29.2%) had a bachelor’s or 4-year college degree, nine of the participants (37.5%) were in the 30–39-year-old age group, 11 participants (47.8%) reported their relationship status as single, never married, 13 participants (54.7%) had private, employer-sponsored health insurance, and six participants (25.0%) had an annual income less than $10,000.

### Cluster Map

**Fig. 2:**
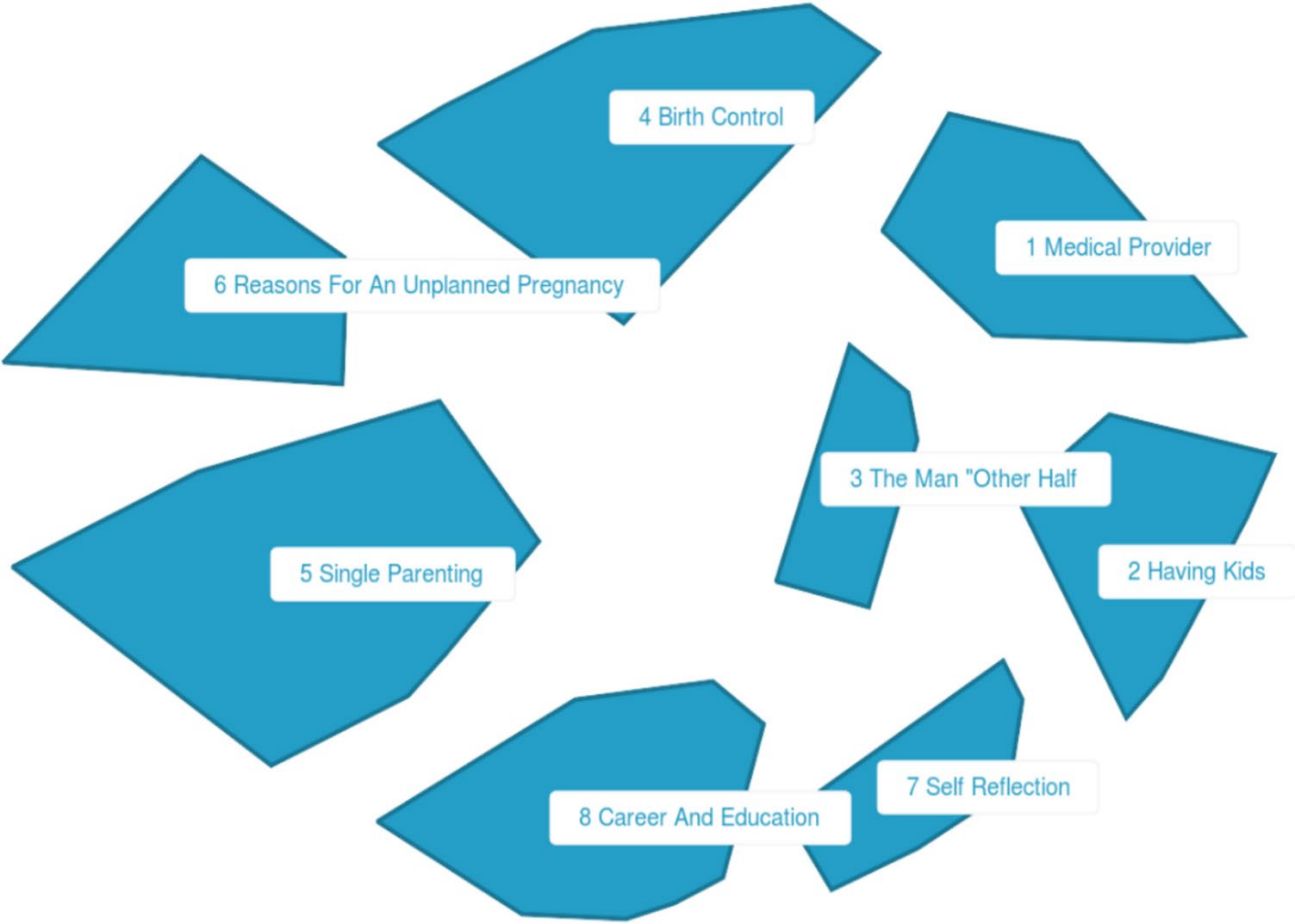
Final cluster map

Each cluster in the cluster map had mapping statements associated with the cluster (see Table 3 and Fig. 2). Each statement rated by the participants in session two was numbered 1 through 102. Through the GCM software’s point mapping process described in Table 2, the statements were grouped into clusters. The eight clusters on the final cluster map are named in Table 3 along with the statements that were grouped into each cluster.

**Table 3:** Clusters and statements

Cluster 1: Medical provider
1. Having older children and not wanting to start over
15. Too many birth control manufacturers—everyone trying to make a dollar
31. Provider refused to tie my tubes
47. Medical provider assumptions about my life
57. Heard horror stories about having kids
59. Health risks/complicated pregnancies
69. Felt supported by medical providers
73. Doing my own research because the OBGYN didn’t educate me
74. Medical provider wanted my partner’s approval/signature for tubal ligation
76. DNA—medical/mental health history (self and partner)
83. Chronic illness and pregnancy/childbirth
Cluster 2: Having kids
2. Birth control fears (developing cancer, horror stories, family/friend died from birth control)
3. Parental advice (just don’t do it, be careful who you lay down with, don’t rush your childhood)
8. When you’re pregnant, you are supposed to be happy
13. Unpleasant experience on state insurance/public assistance
14. Treated differently as a teen mom vs. an adult mom
17. The relationship you have with your medical provider
18. The experience of being pregnant
20. Strong feeling that you are done and don’t want (more) kids
36. Planning a pregnancy so you won’t be surprised
77. Determine if I truly want to have (more) kids
78. Determine if father of baby really wants (more) kids
87. Partner wanted to have a baby
95. Assumptions- once you get married you have kids
Cluster 3: The man “other half”
21. Stress, anxiety/depression
22. Stress being a single mother
55. Pressure from society
61. Having a baby alone (sperm bank)
65. Teasing from friends and family
79. Dating people from other races
Cluster 4: Birth control
4. Violence and/or abuse
16. Time for your body to heal after childbirth
28. Received pushback from providers when wanting to change birth control method
29. Dealing with racism
30. Providers dismissed/overlooked symptoms/concerns
33. Preferring natural methods—pull out method, condoms
42. No tubals offered at religious hospitals
54. I don’t like/want anything foreign in my body (birth control)
63. Giving birth control to teens might promote having sex
75. Medical provider suggested certain birth control methods
84. Celibacy (not having sex)
88. Real/perceived side effects of birth control
89. Birth control is forced on Black women
98. Abstinence
99. Absentee father
100. Abortion as a form of birth control
101. A baby will make him stick around
Cluster 5: Single parenting
5. You don’t think it’s going to happen to you (getting pregnant)
6. Whether your partner will be a good parent or not
7. Where your partner comes from/who they are
11. Want to be done having kids by a certain age
12. Waiting to feel “maternal”
23. Sex feels better without a condom
25. Seen as less driven at work
26. School/education
32. Pressure from family to not be an “old maid”
34. Pray that nothing happens
35. Planning maternity leave so it doesn’t affect your job negatively
37. Past trauma
41. Not a lot of Black men that are in my area that are at my level (education/income)
49. Looking for love
51. Just winged it
64. Gender of baby and/or other children
94. Being 18 years old—now you are an adult and ready for life
96. Allow God to manifest/trusting God
Cluster 6: Reasons for an unplanned pregnancy
24. Settling
43. No planning, it just happened
44. No or poor sex education
45. More Black men in jail, less availability of partners
50. Looked at as sex objects
52. Just having fun, not thinking about getting pregnant
62. Hard to engage with other men outside your neighborhood (segregation)
81. Couldn’t talk about sex with parents
82. Consequences- HIV, other STDs
91. Being under the influence of drugs and/or alcohol
92. Being reckless after being told it would be hard to conceive
Cluster 7: Self reflection
9. Wanting someone to love, so you have a baby
38. My partner
40. Own personal health
46. Mentally/emotionally prepared—“emotional headspace”
48. Marital status
56. Honoring wedding vows
58. Having a healthy relationship
66. Friends
67. Finding myself
72. Emotional intelligence
90. Wanting to better myself
93. Being aware of self wants/needs
97. Age
Cluster 8: Career and education
10. Want to make my parents, grandparents proud
19. Support system (family, friends, medical providers)
27. Responsibilities with other kids
39. Relationship with my parents
53. Just doing what you have to do
60. Having morals, goals, values
68. Financial stability
70. Family acceptance
71. Expense of a child
80. Cultural norms
85. Career-minded
86. Capability to parent
102. Advocate for self without seeming like the “angry Black woman”

### Pattern Match Maps

**Fig. 3:**
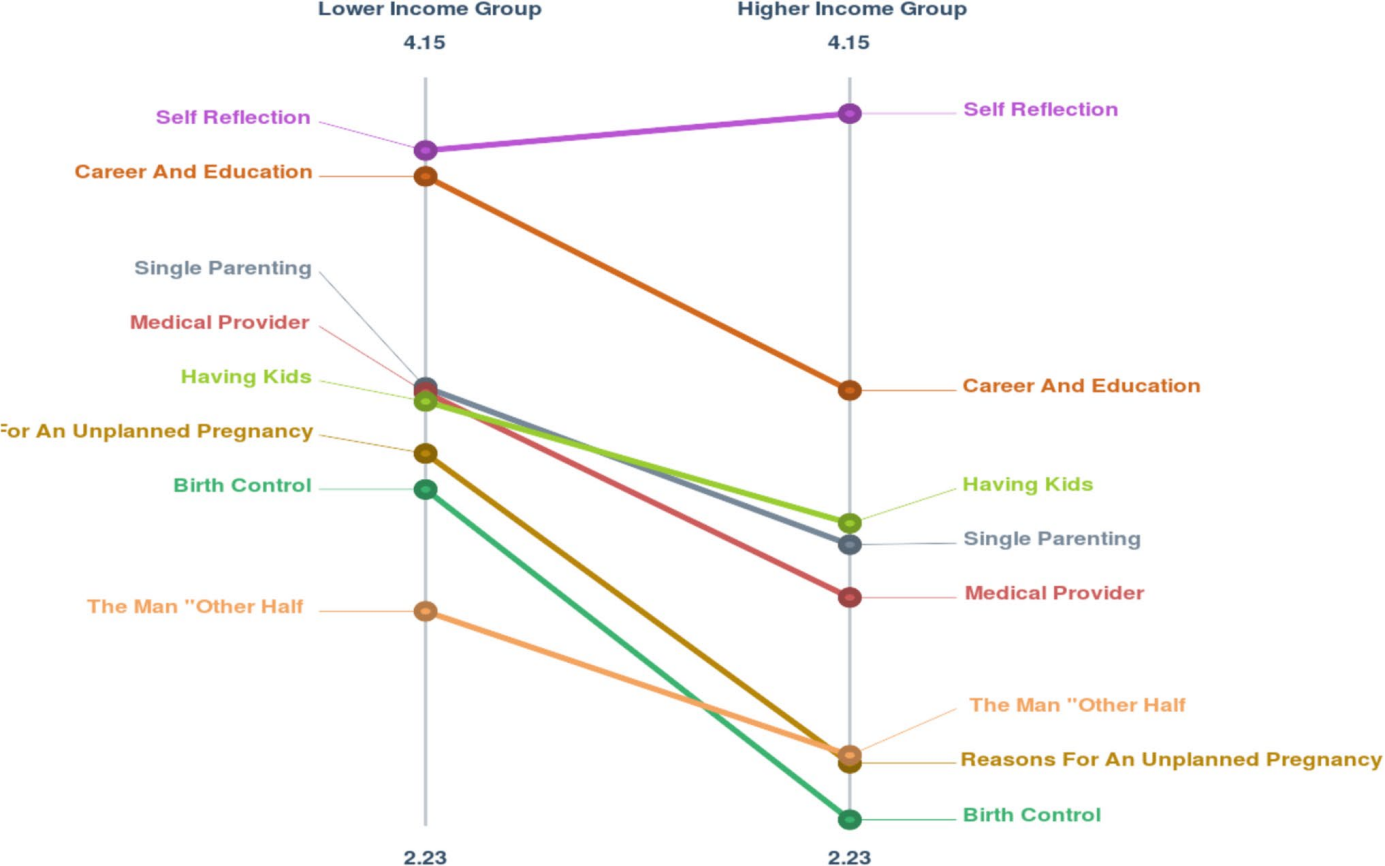
Pattern match visualizing the relative importance of each cluster to each income group in response to rating question 1: How important is each factor in deciding to get pregnant? on a scale of 1 = “relatively unimportant” to 5 = “extremely important”

Pattern match mapping is used to compare subgroups on importance and points out agreements and disconnections [30, 31]. Pattern matches elicit ideas on what the maps mean [31]. The pattern match correlation (correlation coefficient) is the correlation between two patterns in a pattern match. The correlation is computed at a cluster level and indicates the strength of the relationship between the variables on a scale from − 1.00 to + 1.00. The directionality either positive or negative indicates whether the variables are aligned or inverse in their relationship [31]. An example pattern match is displayed in Fig. 3, which shows the average rating (on a scale of 1 = “relatively unimportant” to 5 = “extremely important”) of each cluster by the lower-income group and the higher-income group on separate axes [30, 31]. The pattern match was used to identify information gathered on days 1 and 2 to include information obtained from completed surveys. The groups were analyzed and compared to each other. The patterns indicate the level of importance based on individual ratings by participants and are then aggregated and finally, interpreted by the group. For this study, pattern match compares ratings of the clusters based on income groups. In this pattern match, the lower-income group’s average rating of each cluster of statements is displayed on the axis on the left. The higher-income group’s average rating of each cluster of statements is displayed on the axis on the right. This is an example of an absolute pattern match because the two axes have the same minimum and maximum value. This allows the researcher to understand which cluster of statements were rated, on average, higher or lower importance by each group, and within each group, which clusters of statements were rated higher or lower importance.

Figure 3 visualizes each income group’s average rating of the statements in response to the rating question: How important is each factor in deciding to get pregnant? Both income groups rated the self-reflection cluster highest, followed by the career and education cluster. The cluster called the Man/“Other Half” was rated lowest by the lower-income group, relative to their rating of the other clusters. The higher-income group rated two clusters (reasons for an unplanned pregnancy and birth control) lower than the Man/“Other Half,” but their absolute rating of this cluster was even lower than the lower-income group.

**Fig. 4:**
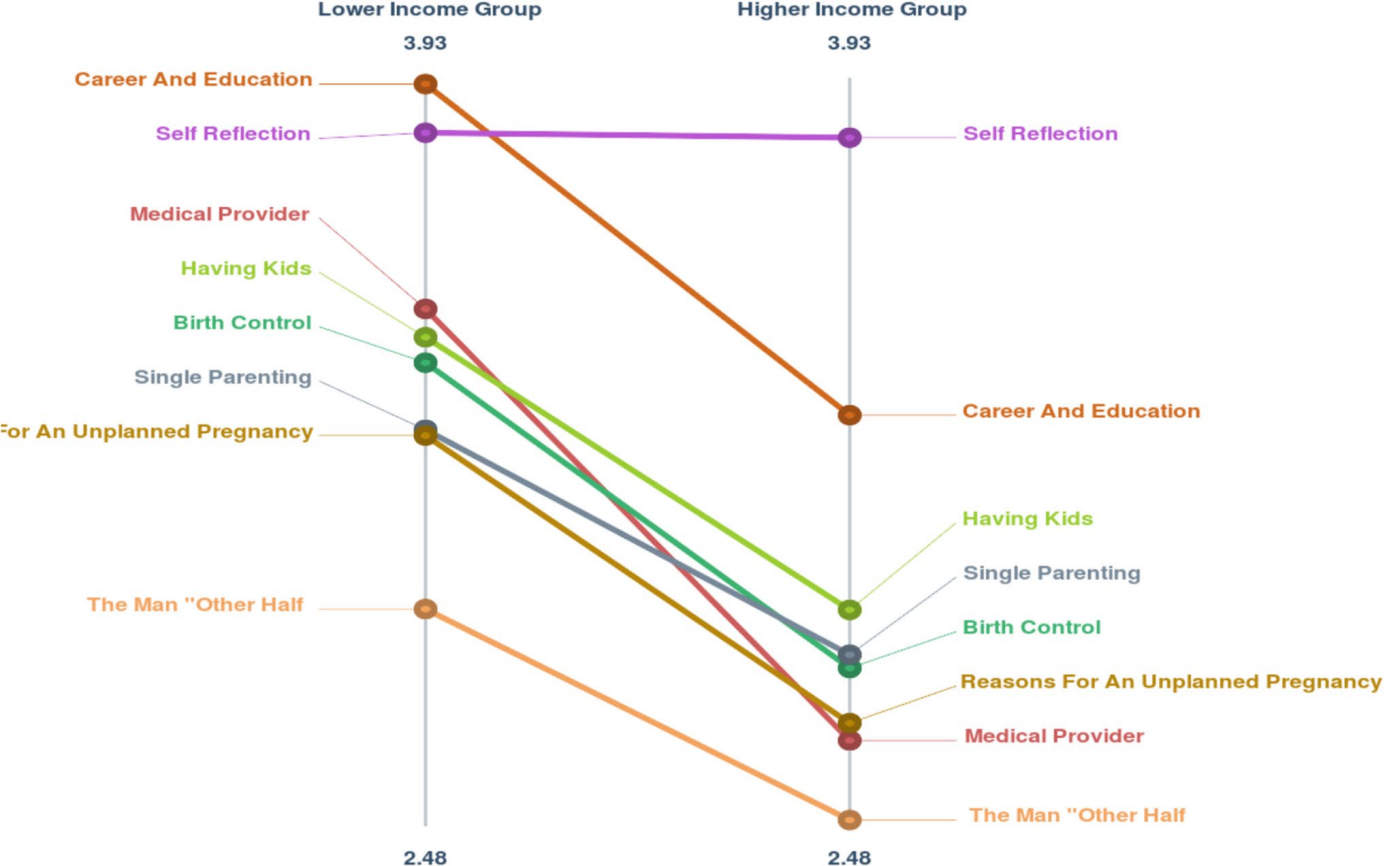
Pattern match visualizing the relative importance of each cluster to each income group in response to rating question 2: How important is each factor in preventing pregnancy? on a scale of 1 = “relatively unimportant” to 5 = “extremely important”

Figure 4 visualizes each income group’s average rating of the statements in response to the rating question How important is each factor in preventing pregnancy? Both income groups rated the self-reflection cluster highly. The lower-income group rated career and education highest, and the higher-income group rated career and education second highest. Both income groups rated the Man/“Other Half” cluster lowest relative to the other clusters, though the higher-income group’s absolute rating of this cluster was lower than the lower-income group’s rating. The lower-income group rated the medical provider cluster third highest, while the higher-income group rated it second lowest.

**Fig. 5:**
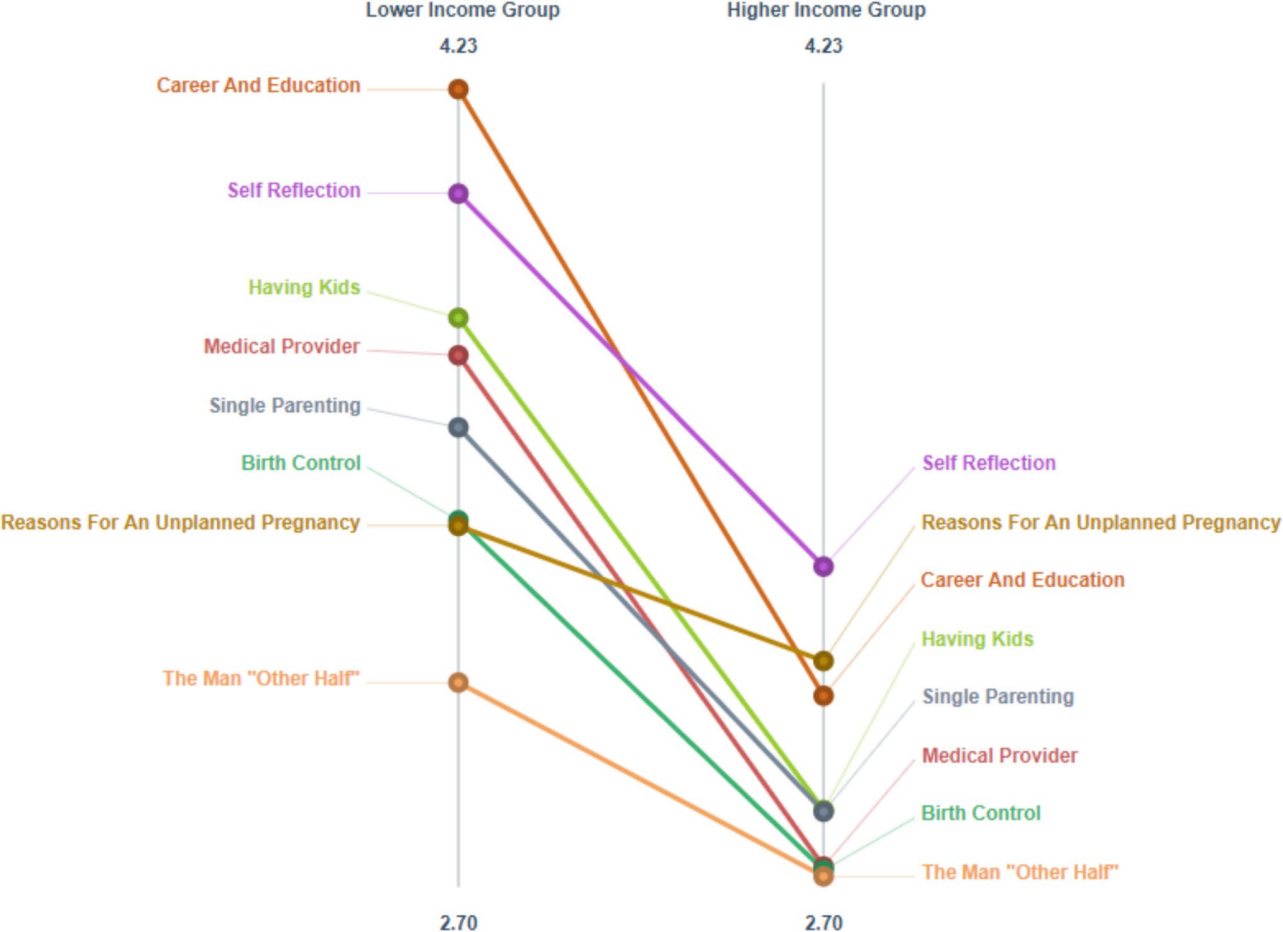
Pattern match visualizing the relative importance of each cluster to each income group in response to rating question 3: How important is each factor in determining if you will use birth control? on a scale of 1 = “relatively unimportant” to 5 = “extremely important”

Figure 5 visualizes each income group’s average rating of the statements in response to the rating question How important is each factor in determining if you will use birth control? The lower-income group had a higher absolute rating for all of the clusters than the higher-income group. In terms of relative rating, both groups rated self-reflection near the top, with this cluster being the most important to the higher-income group and second most important to the lower-income group. The career and education cluster was rated most important to the lower-income group and third most important to the higher-income group. Reasons for an unplanned pregnancy was the second highest rated cluster for the higher-income group, while the lower-income group rated this cluster as their second least important cluster. Both groups rated the Man/“Other Half” as the least important cluster. Birth control was the second least important cluster to the higher-income group, while it was third least important for the lower-income group.

## Discussion

This study identified eight clusters representing 102 ideas that were important to participants when deciding whether to become pregnant or not. Through review of researcher notes from the GCM sessions, the statements generated in the brainstorming and rating steps, and review of the pattern match visualizations, similarities, and differences in the relative and absolute importance of these clusters to the higher- and lower-income groups are evident. From inductive analysis and memoing of these statements, clusters, and pattern matches, three main themes emerged: Inward reflection, relational reflection, and outward reflection, and within each main idea the subtheme of structural racism was apparent [34–36]. Inward reflection included concepts of autonomy, career and education, and the Strong Black Woman (SBW) identity construct. Relational reflection included minimizing the role of the male/partner in deciding pregnancy intention or shared reproductive decision-making. Outward reflection included ideas about how others perceived them in health care settings, including stereotypes, racism, sexism, and classism.

### Inward Reflection

#### Autonomy

When deciding whether to become pregnant, participants in this study consistently maintained that self-reflection and making autonomous decisions were of value to them as individuals. This finding aligns with Aiken and colleagues’ findings that measuring or interpreting a woman’s pregnancy intention is not straightforward [37]. They highlighted the vast range of meanings for women attached to terms such as “planned,” “unplanned,” “intended,” “unintended,” “wanted,” and “unwanted” [37]. While this study’s sample of African American women expressed understanding between the terms planned and unplanned pregnancy, participants did not assign significant value in the terms related to their own experiences and decision-making. For example, in Fig. 5, the higher-income group rated all the clusters that pertain to planned and unplanned pregnancy low (Reasons for an Unplanned Pregnancy, Birth Control, Medical Provider), and the lower-income group rated the Reasons for an Unplanned Pregnancy cluster second to last. In Figs. 3 and 4, Reasons for an Unplanned Pregnancy was one of the lowest rated clusters for both income groups. See Table 3 for the statements within the Reasons for an Unplanned Pregnancy cluster.

Both the lower- and higher-income groups expressed a great deal of autonomy including the decision to take contraceptives, which method was preferred, and where contraceptives could be accessed. Some participants engaged in shared decision-making with their healthcare providers, while others felt coerced to choose the method recommended by the healthcare provider. This was discussed in the brainstorming session with statements from cluster 1: Medical Provider and cluster 4: Birth Control. See Table 3 for the statements that are part of the medical provider and birth control clusters.

Coercive contraceptive practices aimed at women of color have a long history in the USA, so it is not surprising that issues of coercion and autonomy were experienced by the women in this study [38]. African American women specifically have endured systematic attempts to control their fertility [39]. Efforts to reduce unintended pregnancies, especially in low-income and communities of color, have prioritized long-acting reversible contraceptives (LARCs) and focused on addressing cost, and provider and policy barriers to provision. These targeted actions, while well-intentioned, also reduce the autonomy for the women targeted for LARC use: “Studies have shown that existing highly effective contraceptive methods are particularly ill suited to meet the preferences for many women of color” [39]. There is a long history of documented forced sterilization of African American women [40]. The opposite of forced sterilization can happen when the right to bodily autonomy is denied. Both forced sterilization and denial of desired sterilization do not respect the bodily autonomy of African American women. For example, in this study, one participant accessed prenatal and birth care at only faith-based health care facilities and expressed anger that she was not allowed to receive a tubal ligation even though she did not desire more children after her fourth pregnancy. Ironically religious hospitals are often located in predominantly African American, lower-income neighborhoods where residents may not be aware of the religious doctrine of the institution. If Catholic hospitals are the only access point to care in the community, or options are limited due to their insurance plan, then African American women may not receive the full range of reproductive care and contraceptive access.

Participants discussed experiencing what they believed were coercive practices by medical providers, often attempting to push women to accept more contraception or less contraception. For example, in Table 3, in cluster 4: Birth Control, participants’ statements included “received pushback from providers when wanting to change birth control method,” “birth control is forced on Black women,” “providers dismissed/overlooked symptoms/concerns,” and “no tubals offered at religious hospitals.” They shared that despite this perceived coercion, they were able to make decisions that they felt were best for them. This expression of autonomous functioning in the face of coercive practices may suggest some positive movement with respect to the assertion of basic rights among African American women.

#### Career and Education

Rates of African American women entering college have increased in recent decades, but graduation rates remain below that of other racial and ethnic groups. For example, one in five African American women older than 25 years hold bachelor’s degrees, compared with one in two Asian American women and one in three White women, in the same age category [41]. At graduation, African American college graduates owe $7400 more on average than White college graduates. Four years later, the African American to White gap more than triples to $25,000 [42]. This study’s two groups of women (higher and lower income), as illustrated in the group concept mapping clusters, were well aware of how education and socioeconomic status impacts what they are able to provide for future children, but this did not decrease their desire to become pregnant or to continue with a pregnancy that was unplanned.

When considering the importance of pregnancy intention and decision-making, African American women in the lower-income group focused considerably more on career and education than the higher-income group, as illustrated in Figs. 3, 4, and 5.

Twenty-five percent of the lower-income group did not complete high school or a GED; however, 25% of lower-income participants had also achieved a bachelor’s degree or a master’s degree. The lack of secondary education by some group members may be reflective of secondary graduation rates in the city where the study was conducted.

The 4-year graduation rate for Milwaukee Public Schools (MPS) increased from 66.7% in 2018 to 69.1% in 2019 [43]. The graduation rate for MPS has increased steadily in the past five years, beginning in 2015, from 58.2%. The history of segregation in the school system in Milwaukee and lower achievement scores has been recognized for years, especially affecting the African American community in options for post-secondary education.

A 2020 study on the status of African Americans in Milwaukee examined the history and policy behind racial disparities [44]. This examination included income, employment, hiring practices, incarceration, education, health, and home ownership. The study found that 72.2% of African American students attended hyper-segregated schools, the highest rate in the USA. This rate is about the same as the rate in 1965 [44]. Residential segregation has been closely related to socioeconomic disparities between African Americans and White people. In the US economic disparities were imbued historically by discriminatory institutional policies [45]. Segregation relates to greater African American-White disparities in school quality, employment opportunities, and individual health issues [45]. Specific to Milwaukee, segregation imposes disparities in health, education, income, and employment that result in cyclical disadvantages for African American families, thereby the most vulnerable in the community make reproductive decisions from their worldview, often which is generational, influenced by micro- and meso- systems.

The higher-income group participants achieved more educational success, with 75% obtaining a bachelor’s or master’s degree. The higher-income group also reported much higher household incomes from their professional positions. The higher-income group explicitly expressed the pressure of the Strong Black Woman construct, lack of available African American men for committed relationships, balancing career and family, and career trajectory. In session 3, when participants interpreted the data, the participants discussed the statements related to Strong Black Woman, challenges in finding relationships with African American men, balancing career and family, and focus on career path in cluster 3: The Man/“Other Half,” cluster 5: Single Parenting, Cluster 6: Reasons for an Unplanned Pregnancy, Cluster 7: Self Reflection, and Cluster 8: Career and Education (see Table 3). As illustrated in Fig. 3, the Career and Education cluster was ranked second highest for both income groups, with several participants sharing they were pursuing more education or thinking about it for career advancement.

Research on educational attainment focuses more on non-completion, rather than on larger structural issues that address race, class, and gender inequities in the larger society [41]. A gap in the literature could be filled with more attention to the intersectional reasons for lack of educational attainment. The finding that both income groups rated highly statements related to career and education when deciding to get pregnant (Fig. 3) and when deciding to prevent pregnancy (Fig. 4) raises important questions about how the lack of educational opportunities affect the reproductive decision-making among African American people. Educational attainment emerged unexpectedly. This is important in two different ways. Some participants wanted to pursue further education and career opportunities, which served as an incentive for delaying pregnancy. Women who had children pursued educational and career opportunities to take care of their children. This was identified as an important component to their identity and motherhood. While women in this study recognized the importance of education in their lives, the reality is for some, their education did not prepare them for success.

#### Strong Black Woman

Researchers have begun studying the stereotype of the Strong Black Woman (SBW) related to many topics. The SBW is well known in the African American community and is a long held cultural strength [28]. The higher-income participants brought up this construct while brainstorming in session one. During the discussion across all three sessions, participants from both income groups brought up, “having to push through when something happens.” This idea is also reflected in statements in cluster 8: Career and Education. See the statements from cluster 8 in Table 3.

Compounded by the harsh realities of intersectional oppression brought on by the enslavement of African people, the SBW schema reinforced cultural expectations of extraordinary resilience, independence, and strength [28, 48]. Despite the advantages the SBW schema affords individuals when coping with challenges, individuals who strongly internalize these high expectations are at risk for premature health deterioration, including negative physical and mental health outcomes [19]. The participants expressed the stress of the expectation and necessity to take on the attributes of a SBW. For example, in the brainstorming sessions, participants said, “pressure from mom for the ‘real world’/coming of age,” “how your daughter turns out is a reflection of the mother,” “pressure from all angles,” “use a Black woman for anything,” “Black women are pushed harder and held to higher standards than Black men are,” and “I don’t want my daughter to be dependent on a man.” This suggests cultural and family expectations that participants in this study were expected to develop and maintain the essence of a Strong Black Woman. This schema may have reinforced their independence and autonomy when choosing birth control methods and making reproductive decisions. It also may have reinforced feelings of depression, stress, and trauma.

The response of African American women to adversity is varied and includes personal coping practices, as well as cultural strength, wisdom, and healing that are reflective of their community [46]. The pressure, not necessarily the desire, to maintain being a SBW and “pushing through” affects every area of an African American woman’s life. The need to “push through” minimizes women’s need to attend to the stress, depression, and trauma in their lives. Because these issues intersect, this is not always transparent and cannot be operationalized discretely and separately by individual women.

Women in the higher-income group also discussed navigating White spaces in higher education, employment, housing, and a variety of social spaces, and the resulting stress of navigation in these spaces that were never intended for them was ever-present [47, 48]. Their expression of their feelings in the group sessions indicates they do deal with stress, depression, and trauma, but perhaps it is so normalized in their experience that they do not specifically name these feelings as such when asked specifically about them. For example, they also expressed feelings of “having to push through when something happens,” just as the lower-income group did.

### Relational Reflection

Reflection on the more relational aspects of pregnancy intention and reproductive decision-making was a major part of the GCM process in both the higher and lower-income groups. Both groups indicated through brainstorming that they did not place a high value on shared decisions regarding contraceptives, pregnancy intention, or reproductive decision-making with a sexual partner. Both groups rated the Man/“other half” cluster lowest of the clusters in response to the question “How important is each factor in preventing pregnancy?” In the Man/“Other Half” cluster, discussion and naming of stress, anxiety, and depression came up. For example, statement 21: “Stress, anxiety/depression,” statement 22: “Stress being a single mother,” and statement 55: “Pressure from society” were statements present in cluster 3: the Man/“Other Half” (see Table 3). Participants also discussed the stress of being a single mother and being judged for either having children or not having children, as evident in Table 3, where participants brainstormed statements 22: “Stress of being a single mother” and 61: “Having a baby alone” and grouped them in cluster 3: the Man/“Other Half.” These statements indicate that the women in this sample were concerned about pregnancy and being solely responsible for a child. They acknowledged stress and uncertainty about the role their sexual partner will take if they become pregnant, and therefore felt they must take control of their fertility.

In 2014, Abrams and colleagues examined the SBW schema and found that African American women in their study, expressed feelings of oppression and the limited socioeconomic mobility of African American men, forcing the women to feel that they must be the leader in their family to ensure survival of their families and their communities [28]. The African American father’s role in the family has been more deleteriously affected by institutionalized racism than any other racial or ethnic group, which has obvious consequences for African American women who bear more family responsibilities [49]. Further research into the presence and absence of African American men in the context of African American women’s reproductive decision-making is warranted.

It is difficult to talk about the role of a romantic or sexual partner in a study on reproductive decision-making in African American women without framing the status of African American men. Most participants expressed preference for dating/marrying African American men, but some women from the higher-income group openly discussed whether they should contemplate dating outside of their race or pursue fertility assistance through a sperm bank, because of their perceived lack of datable African American men. Our society lacks broad social supports for children and families, leaving individual women and their partners to shoulder the costs and responsibilities of raising children. As heard during participant conversation, the women expressed the need to have a partner who can contribute to these costs and responsibilities as one element of a desirable partner in reproductive decision-making. Levine’s 2020 study found that 17.7% of African American males in Milwaukee make more than $40,000 a year, compared to 46.3% of White males. The statistic is even bleaker for African American women, with only 14.6% making over $40,000 [44]. The earnings of African American males in Milwaukee demonstrate the worst disparity in the nation with African American males earning only 59.7% of White male workers. Remarkably in Milwaukee, the annual median African American household income in 1979 was 58.3% of a median White household. In 2018, the figure had fallen to 42%. Across the nation the median annual income for African Americans had risen to 60.9% of the median White household [44].

Across the USA, African American people are incarcerated at much higher rates than White people. In Wisconsin, African Americans had the third-highest incarceration rate out of all 50 states [44]. In Wisconsin, one in 20 African American men are incarcerated, giving Wisconsin the tenth highest African American male incarceration rate in the country [50]. Wisconsin is one of the worst states for African American incarceration. These institutional structures negatively impact the African American family, making marriage and two-parent households less attainable, if desired. The study participants expressed having to learn to do everything themselves and to keep pushing for whatever outcome they desired. The stereotype of the SBW serves to oppress African American women as it embraces the idea that somehow African American women are super-human and able to withstand violence, misogyny, racism, and other oppression that they were historically forced to endure since African enslavement.

### Outward Reflection

In 2017, Gadson and colleagues analyzed experiences of racism and discrimination in the health care systems’ prenatal care utilization and found that over 40% of participants reported communication issues during prenatal care, 24% perceived discrimination during hospitalization at birth [51]. African American and Hispanic women were associated with nearly three times higher odds of discrimination due to race, language, or culture [51]. Uninsured women had nearly twice the odds of experiencing any perceived discrimination by race-based and insurance-based discrimination [51]. Perinatal quality metrics have been endorsed by the Centers for Medicaid and Medicare Services and require hospitals to report on quality metrics related to care. It is unknown whether racial and ethnic disparities related to care will be included in this data [52]. African American middle-class women may have more exposure to conscious and unconscious bias as they navigate White spaces and are exposed to more and different discrimination than lower-income African American women living in segregated communities.

Data show that African American women who are dying of pregnancy-related causes are younger, less educated, and more likely to be unmarried [52]. This was not the case for the participant in the higher-income group who shared her story about pregnancy-related hypertension, or many other African American women in the news media, including Shalon Irving, a Lieutenant Commander in the Public Health Service who died from complications of high blood pressure, and Kira Dixon Johnson who died in 2016 after complications of a scheduled cesarean section. These women were educated, upwardly mobile, and had experience accessing the health system. Another mainstream example is Serena Williams, a wealthy, famous, well-connected woman with a White husband with affluence, who had access to the best medical care, but still experienced dangerous post-birth complications with blood clots. Despite being of affluent means, strongly advocating for themselves, and knowing something was wrong, these women were not listened to by health care providers and suffered adverse consequences to their health. The women in this study sample who are not as affluent or well-known must continue to practice autonomy and advocate for themselves, due to the burden of structural determinants.

Participants reflected on the care and treatment they received when they were covered by Medicaid. One participant experienced feelings of not being heard and being treated as if she was drug-seeking when she advocated for herself, due to what turned out to be postpartum eclampsia. She advocated for herself, knowing something was wrong, but was not listened to by health care providers. Luckily, her advocacy, she believes, saved her life.

### Limitations

This study relied on a small convenience sample of African American women in a medium-sized midwestern city and is not representative of African American women across the country. Additionally, the inclusion criteria for this study did not include adolescents, foreign born women who are Black, transgender people, or people over the age of 44. Study findings could differ among people who did not meet these inclusion criteria. Future research may also consider recruiting participants within narrower income ranges to enhance the ability to compare decision-making factors within smaller income groups. GCM is a resource intensive method. It requires time, multiple sessions, and multiple staff. Additionally, each cluster’s name is a simple representation of more complex statements within each cluster, and the analysis should take into consideration the individual statements within each cluster because they provide richer insight into the ideas present.

### Strengths

Despite the GCM limitations, this approach was also a main strength of this study, because the method is participatory, privileges the participant voice, and generates a multi-layered method of inquiry and analysis. Another strength of this study was the diverse characteristics of the sample. There was diversity in marital and relationship status, sexuality, age, income level, and education among participants. The sample also varied in history of pregnancy intent, number of pregnancies, contraceptive choice, and reproductive decision-making. The richness of this data was solicited from group discussion and individual responses. The three-day group process itself may have been therapeutic for the participants, who were able to share challenging lived experiences within the group setting.

Finally, the group concept mapping method allowed for the intersectionality of gender, race, and class to be expressed in the data analysis. This was an important strength of this method, allowing for this complexity to exist, because this parallels the complexity of African American women’s lives when making reproductive decisions. Furthermore, group concept mapping is a stronger methodological approach for understanding complex phenomenon, such as family planning in African American women’s lives grounded in the theoretical frameworks of intersectionality and critical race theory, than focus groups or in-depth interviews. GCM could be used to enhance more traditional qualitative and mixed methods approaches.

### Implications for Public Health and Health Care

Findings from this study provide public health practitioners and researchers with a nuanced understanding of the complexities of intersectional identities and lived experiences that influence pregnancy intention and reproductive decision-making within a sample of women from a population that has been marginalized for centuries. Pattern match mapping shows the higher-income group has ranked all the clusters lower than the lower-income group in response to the question, “How important is each factor in determining if you will use birth control?” The raw data from the higher-income group’s brainstorming session includes insight into the unique pressure higher-income African American women face when making reproductive decisions. Statements like “Being a single mother (the pressure to be both parents),” “stereotypes about Black women,” “Pressure from all angles,” “racism (overall theme),” and “Black women are pushed harder and held to higher standards than Black men” show there are complexities to reproductive decision-making at the intersections of race, gender, and class status. This marginalization and outright discrimination have affected African American women in every area of their lives and reflects how their decision-making and pregnancy intention may have been shaped.

Pregnancies do not always fit into planned/unplanned, wanted/unwanted, or intended/unintended binaries. Rather pregnancy experiences exist within spectrums of experiences based on individual level identities (e.g., race, gender, class status, health history), structural factors (e.g., White supremacy, compulsory heterosexuality, capitalism, institutional hegemonies), and other contextual factors named by participants in the brainstorming process, that should be acknowledged and understood by health care providers, birth workers, human services providers, and public health practitioners in order to provide salient and responsive services to pregnant people. This includes the acknowledgement and understanding of past and present coercive reproductive health care practices, which should be integrated into practice, social media campaigns, and health education. Public health practitioners can facilitate key messages to the public related to reproductive health, all-options for birth control, becoming pregnant and terminating a pregnancy if desired. Participants remarked that birth control and sterilization decisions were pushed upon them by medical providers, but they lacked support and guidance in general, and reproductive health knowledge, care related to fertility, and the autonomy to advocate for themselves. Public health and health care practitioners should consistently present all birth control and reproductive care options and use of the teach-back method to ensure patient understanding [53]. Follow-up telephone calls to reinforce teaching and openness to answer all reproductive health questions could build trust and rapport between the public health practitioner and the patient. The need for multi-level interventions for culturally specific care and advocacy led by African American women cannot be overstated. The principles of social justice, asserting the right to have children, not have children and to parent children is fundamental in the care and treatment of African American people in the USA.
